# Dietary nitrate‐induced increases in human muscle power: high versus low responders

**DOI:** 10.14814/phy2.13575

**Published:** 2018-01-25

**Authors:** Andrew R. Coggan, Seth R. Broadstreet, Deana Mikhalkova, Indra Bole, Joshua L. Leibowitz, Ana Kadkhodayan, Soo Park, Deepak P. Thomas, Dakkota Thies, Linda R. Peterson

**Affiliations:** ^1^ Departments of Kinesiology Indiana University Purdue University Indianapolis Indianapolis Indiana; ^2^ Cellular and Integrative Physiology Indiana University Purdue University Indianapolis Indianapolis Indiana; ^3^ Departments of Radiology Washington University School of Medicine St. Louis Missouri; ^4^ Department of Medicine Washington University School of Medicine St. Louis Missouri

**Keywords:** Fiber type, isokinetic dynamometry, nitric oxide, sex differences

## Abstract

Maximal neuromuscular power is an important determinant of athletic performance and also quality of life, independence, and perhaps even mortality in patient populations. We have shown that dietary nitrate (NO_3_^−^), a source of nitric oxide (NO), improves muscle power in some, but not all, subjects. The present investigation was designed to identify factors contributing to this interindividual variability. Healthy men (*n* = 13) and women (*n* = 7) 22–79 year of age and weighing 52.1–114.9 kg were studied using a randomized, double‐blind, placebo‐controlled, crossover design. Subjects were tested 2 h after ingesting beetroot juice (BRJ) either containing or devoid of 12.3 ± 0.8 mmol of NO_3_^−^. Plasma NO_3_^−^ and nitrite (NO_2_^−^) were measured as indicators of NO bioavailability and maximal knee extensor speed (*V*_max_), power (*P*_max_), and fatigability were determined via isokinetic dynamometry. On average, dietary NO_3_^−^ increased (*P* < 0.05) *P*_max_ by 4.4 ± 8.1%. Individual changes, however, ranged from −9.6 to +26.8%. This interindividual variability was not significantly correlated with age, body mass (inverse of NO_3_^−^ dose per kg), body mass index (surrogate for body composition) or placebo trial *V*_max_ or fatigue index (in vivo indicators of muscle fiber type distribution). In contrast, the relative increase in Pmax was significantly correlated (*r* = 0.60; *P* < 0.01) with the relative increase in plasma NO_2_^−^ concentration. In multivariable analysis female sex also tended (*P* = 0.08) to be associated with a greater increase in Pmax. We conclude that the magnitude of the dietary NO_3_^−^‐induced increase in muscle power is dependent upon the magnitude of the resulting increase in plasma NO_2_^−^ and possibly female sex.

## Introduction

Maximal neuromuscular power is an important determinant of athletic performance and is also highly significant from a clinical perspective, as reductions in power contribute to impaired quality of life, disability, and possibly even mortality in various patient populations (e.g., the elderly (Guralnik et al. 1994), heart failure (HF) patients (Hülsmann et al. 2004)). It is therefore noteworthy that recent studies have demonstrated that acute or chronic supplementation with dietary nitrate (NO_3_^−^), a source of nitric oxide (NO) via the enterosalivary pathway (Lundberg and Weitzberg 2009), can influence muscle contractile properties (Haider and Folland 2014; Coggan et al. 2015a,b; Justice et al. 2015; Rimer et al. 2016; Whitfield et al. 2017). In a previous study, for example, we found that acute ingestion of NO_3_^−^ increased maximal knee extensor speed and power in healthy, untrained individuals by 11 and 6%, respectively, (Coggan et al. 2015a). We observed a similar dietary NO_3_^−^‐induced enhancement of maximal neuromuscular power in athletes (Rimer et al. 2016), and an even greater increase (i.e., 13%) in patients with HF (Coggan et al. 2015a). NO_3_^−^ (or nitrite (NO_2_^−^)) ‐induced improvements in muscle contractility have also been observed in some (Haider and Folland 2014; Justice et al. 2015; Whitfield et al. 2017), albeit not all (Hoon et al. 2015), recent studies of voluntary or electrically stimulated isometric exercise.

Based on these previous studies (Haider and Folland 2014; Coggan et al. 2015a,b; Justice et al. 2015; Rimer et al. 2016; Whitfield et al. 2017), it therefore appears that dietary NO_3_^−^ can enhance the inherent contractile properties of human muscle. However, as with NO_3_^−^‐induced improvements in endurance performance (Christensen et al. 2013; Boorsma et al. 2014) (or reductions in blood pressure (Kapil et al. 2010)), not all individuals seem to respond equally. Specifically, only about three‐fourths of the subjects we have studied previously have demonstrated improvements in muscle speed and/or power with NO_3_^−^ intake. The reason for this variability between individuals is not clear, but it may be related to the extent to which NO_3_^−^ intake increases NO bioavailability. This hypothesis is suggested by the key role played by oral bacteria in reducing ingested NO_3_^−^ to NO_2_^−^, the immediate precursor for NO synthesis via the enterosalivary pathway (Lundberg and Weitzberg 2009). Alternatively and/or in addition, based in part on animal studies it has been proposed that the effects of dietary NO_3_^−^ supplementation are greatest in type II, or fast‐twitch, muscle fibers (Jones et al. 2016). The interindividual variability in muscle power improvements that we have observed therefore may be related to differences in muscle fiber type distribution.

The purpose of this study was to test the hypothesis that interindividual differences in the effects of dietary NO_3_^−^ on muscle function are related to interindividual differences in NO production and/or in the percentage of fast‐twitch muscle fibers. To do so, we determined the relationship between changes in muscle power due to NO_3_^−^ ingestion and markers of NO bioavailability (i.e., plasma NO_3_^−^ and NO_2_^−^ levels) and muscle fiber type (i.e., maximal knee extensor velocity (Vmax) and fatigability in the absence of NO_3_^−^ intake) in a heterogeneous group of healthy men and women. We recruited subjects widely varying in other characteristics (e.g., age) as well, to determine whether there was any relationship between such factors and the response to dietary NO_3_^−^. The results of this study provide insight into the mechanisms responsible for interindividual differences in the effects of NO_2_^−^ supplementation on muscle power, which may prove useful in optimizing this intervention in both athletes and clinical populations.

## Methods

### Subjects

We studied 13 men and 7 women ranging in age from 22 to 79 (mean 47 ± 20) years, in body mass from 52.1 to 114.9 (mean 78.2 ± 16.3) kg, and in body mass index (BMI) from 19.1 to 32.6 (mean 25.8 ± 4.2) kg/m^2^. All of the subjects were healthy, based upon medical history, physical examination, and standard blood chemistries. Although all were normally active, only two exercised regularly, and none were engaged in training for competitive sports. None of the subjects smoked. Additional exclusion criteria included use of drugs that can block reduction of NO_3_^−^ and NO_2_^−^ to NO (i.e., prescription sex hormones, antacids, proton pump inhibitors, or xanthine oxidase inhibitors) (Lundberg et al. 1994; Obach et al. 2004) or can potentiate the effects of the latter (i.e., phosphodiesterase inhibitors) (Webb et al. 1999). Women who were pregnant or lactating were also excluded. Approval for the study was obtained from the Human Subjects Office at Indiana University and the Human Research Protection Office at Washington University School of Medicine, and all subjects provided written, informed consent. Partial data from some of these subjects has been presented previously (Coggan et al. 2015b).

### Experimental design and protocol

Each subject was studied twice using a double‐blind, placebo‐controlled, randomized design. During one trial, subjects were tested after ingesting 140 mL of a commercial beetroot juice (BRJ) supplement (Beet It^®^, James White Drinks, Ipswich, UK) containing 12.3 ± 0.8 mmol of NO_3_^−^. During the other trial, they ingested an equal volume of concentrated BRJ from which the NO_3_^−^ had been removed by the manufacturer. A washout period of 1–2 weeks separated the two trials (i.e., NO_3_^−^ vs. placebo). Since use of an antibacterial mouthwash, tooth brushing, or chewing gum can block the conversion of NO_3_^−^ to NO_2_^−^ by oral bacteria (Lundberg et al. 1994; Govoni et al. 2008), subjects were instructed to avoid these behaviors on study days. They were also instructed to avoid high NO_3_^−^ foods throughout the study, with adherence to this instruction verified by analysis of food records by a dietician.

Subjects arrived at the Clinical Research Unit in the morning after avoiding food, caffeine, or alcohol intake for the previous 12 h. A catheter was first inserted in an antecubital vein and a blood sample obtained for subsequent measurement of plasma NO_3_^−^ and NO_2_^−^ concentrations via high‐performance liquid chromatography (ENO‐30, Eicom USA, San Diego, CA). These measurements were repeated and 2 h of quiet rest, after which the contractile properties of the knee extensor muscles of the subject's dominant leg were determined using an isokinetic dynamometer (Biodex System 4 Pro, Biodex Medical Systems, Shirley, NY) as previously described (Coggan et al. 2015a,b). Briefly, each subject performed 3–4 maximal knee extensions at angular velocities of 0, 1.57, 3.14, 4.71, and 6.28 rad/sec, with 2 min of rest between each set. The resulting torque data were filtered and smoothed to eliminate artifacts, after which peak power was calculated by multiplying the peak torque observed at each velocity by that velocity. The power‐velocity data were then fit with a parabolic function to determine the subject's *V*_max_ and maximal power (*P*_max_). After an additional 2 min of rest, the subject performed 50 consecutive maximal knee extensions at an angular velocity of 3.14 rad/sec to determine their resistance to fatigue (i.e., fatigue index, =% decrease in power from first 1/3 to last 1/3 of the test) during repeated muscle contractions. Following a 10 min rest period, the final plasma samples were obtained after which the subject was fed a light meal and released.

### Data analysis

Statistical analyses were performed using GraphPad Prism version 7.02 (GraphPad Software, La Jolla, CA). Normality of data distribution was first tested using the D'Agostino‐Pearson omnibus test. Data from the placebo and NO_3_^−^ trials were subsequently compared using two‐way (treatment x order) ANOVA, with subject as a repeated measures factor within treatment. Intraclass correlation coefficients were calculated from the ANOVA results to quantify the reliability of the data. Standard Pearson product correlations were calculated to explore the relationship between relative changes in Pmax as the dependent variable and sex, age, body mass (inverse to NO_3_^−^ dose in *μ*mol/kg), BMI, placebo trial *V*_max_, placebo trial fatigue index (in vivo indicators of muscle fiber type distribution), percent change in plasma NO_3_^−^, or percent change in plasma NO_2_^−^ as independent variables. The overall false discovery rate was limited to 10% using the Benjamini–Hochberg procedure. As this was an exploratory study, stepwise forward regression was also employed using the same dependent and independent variables, with the *P* value to enter the model similarly set to 0.10.

## Results

**Figure 1:**
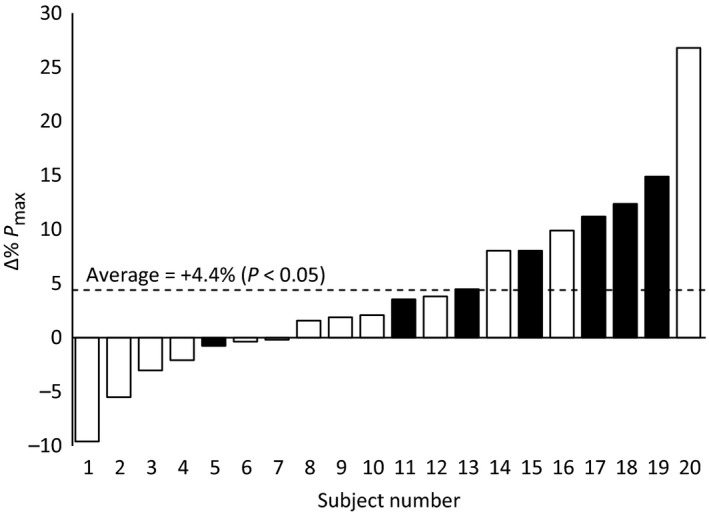
Individual relative changes in maximal knee extensor power (*P*_max_) in response to dietary NO_3_^−^ intake. *Open bars*, male subjects. *Closed bars*, female subjects. The overall average response is also shown (*dashed line*).

The effects of ingesting BRJ without or with NO_3_^−^ on plasma NO_3_^−^ and NO_2_^−^ concentrations are shown in Table 1. No significant changes occurred in the placebo trial, whereas during the NO_3_^−^ trial, both NO_3_^−^ and NO_2_^−^ increased significantly. This was accompanied by a significant (*P* < 0.05) elevation in Vmax, which increased from 12.3 ± 2.5 (range: 9.3–20.2) rad/sec in the placebo trial to 13.2 ± 3.1 (range: 8.4–20.2) rad/sec in the NO_3_^−^ trial. Pmax also increased significantly (*P* < 0.05), that is, from 6.3 ± 2.3 (range: 2.8–10.7) to 6.6 ± 2.4 (range: 2.7–11.8) W/kg. Individual changes varied from −9.6 to +26. % (Fig. 1). On the other hand, the fatigue index was unaltered by NO_3_^−^ intake, averaging 61 ± 13 (range: 34–78) and 61.6 ± 13.5 (range: 32–78)% during the placebo and NO_3_^−^ trials, respectively. All three performance measures were highly reliable, with intraclass correlation coefficients of 0.94, 0.98, and 0.89 for *V*_max_, *P*_max_, and fatigue index, respectively. No adverse effects were observed. These observations confirm and extend our previous findings (Coggan et al. 2015a,b; Rimer et al. 2016). The remainder of our effort therefore focused upon attempting to elucidate the factors responsible for the marked variability between subjects in the response to NO_3_^−^ intake.

**Table 1:** Changes in plasma NO_3_^−^ and NO_2_^−^ in response to NO_3_^−^

		Time point			
	Trial	Pre	1 h	2 h	10 min post
Plasma NO_3_^−^ (*μ*mol/L)	Placebo	26 ± 11	23 ± 9	22 ± 7	23 ± 11
	Nitrate	30 ± 18	334 ± 111†	351 ± 74†	346 ± 91†
Plasma NO_2_^−^ (*μ*mol/L)	Placebo	0.29 ± 0.22	0.30 ± 0.26	0.30 ± 0.28	0.29 ± 0.36
	Nitrate	0.36 ± 0.40	0.44 ± 0.33*	0.47 ± 0.34†	0.57 ± 0.32†

Values are mean ± SD for *n* = 19.

Nitrate trial significantly higher than Placebo trial at same time point: *P < 0.01, †*P* < 0.0001.

**Figure 2:**
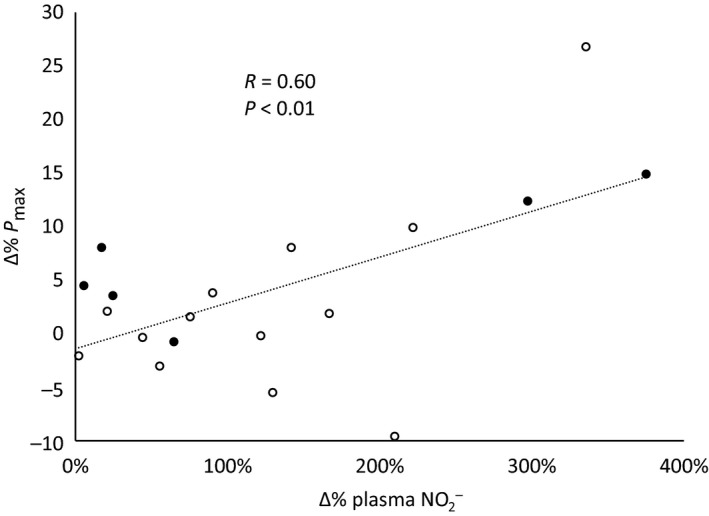
Relationship of relative changes in maximal knee extensor power (*P*_max_) to relative changes in plasma NO_2_^−^ concentration in response to dietary NO_3_^−^ intake. *Open symbols*, male subjects. *Closed symbols*, female subjects. Plasma samples from one female subject were not available for analysis; data for the remaining 19 subjects are therefore shown.

In univariable analyses, the relative increase (i.e., Δ%) in Pmax due to dietary NO_3_^−^ ingestion was not significantly correlated with sex, age, body mass, or BMI, or with placebo trial Vmax or fatigue index (Table 2). The relative magnitude of the increase in *P*_max_ was also not correlated with the relative change in plasma NO_3_^−^ concentration (Table 2). There was, however, a significant correlation between the relative increase in Pmax and the relative increase in plasma NO_2_^−^ concentration due to NO_3_^−^ intake (Table 2; Fig. 2). The relative change in plasma NO_2_^−^ concentration was also the strongest predictor of relative changes in *P*_max_ in the multivariable analysis (Table 3). Female sex also tended to be a positive predictor of relative increases in Pmax in the multivariable analysis (Table 3). In keeping with this, female subjects tended (i.e., *P* = 0.06 by Fisher's exact test) to be more likely to exhibit a greater‐than‐average increase in Pmax, that is, to be “high responders” (Fig. 2). Taken together, the relative change in plasma NO_2_^−^ concentration and subject sex explained ~40% of the interindividual variation in the effect of NO_3_^−^ intake on muscle power (i.e., *R* ^2^ of multivariable regression = 0.38).

**Table 2:** Pearson‐product correlation coefficients between Δ% Pmax and potential explanatory variables

Sex	Age	Body mass	BMI	Placebo *V*_max_	Placebo fatigue index	Δ% NO_3_^−^	Δ% NO_2_^−^
0.31	−0.16	−0.16	0.10	0.08	−0.25	−0.05	0.60*

*P < 0.01.

**Table 3:** Results of stepwise forward regression

Predictor	Beta coefficient	SE	Lower 95% CI	Upper 95% CI	*t*	*P*
Δ% NO_2_^−^	0.038	0.014	0.011	0.064	2.81	0.005
Sex	0.056	0.032	−0.006	0.118	1.76	0.079

## Discussion

The purpose of this study was to identify (if possible) factors contributing to interindividual variability in improvements in muscle contractile function resulting from dietary NO_3_^−^ intake. Based on previous research, we hypothesized that such differences would be related to differences between individuals in markers of NO bioavailability and/or muscle fiber type. Consistent with the first hypothesis, we found a significant correlation between the relative increase in *P*_max_ and the relative increase in plasma NO_2_^−^ concentration due to NO_3_^−^ ingestion. Our second hypothesis, however, was not supported, as there was no association between the increase in *P*_max_ and baseline *V*_max_ or fatigue index, in vivo indicators of muscle fiber type distribution (see below). Finally, our data provide preliminary support for the novel hypothesis that, at least in terms of improvements in maximal neuromuscular power, women are more likely than men to benefit from dietary NO_3_^−^ supplementation.

Reduction in NO_3_^−^ to NO_2_^−^ by oral bacteria plays a critical role in the production of NO via the enterosalivary pathway (Lundberg et al. 1994; Govoni et al. 2008; Lundberg and Weitzberg 2009). In fact, this step appears to be possibly rate‐limiting, as demonstrated by the much smaller increase in plasma NO_2_^−^ versus NO_3_^−^ following NO_3_^−^ ingestion (Table 1). Accordingly, previous studies have observed a significant correlation between the magnitude of the increase in plasma NO_2_^−^ following NO_3_^−^ ingestion and the improvement in endurance performance ability (Wilkerson et al. 2012; Hoon et al. 2014). Our results are similar, as we found that interindividual differences in how much plasma NO_2_^−^ concentration was elevated by NO_3_^−^ intake accounted for about one‐third of the variation between individuals in the increase in Pmax. It is possible that this significant correlation reflects a direct effect of NO_2_^−^ on muscle contractility. Indeed, in cardiac muscle NO_2_^−^ has been shown to nitrosylate cysteine residues of various membrane proteins independently of NO (Montesanti et al. 2014). In skeletal muscle, however, S‐nitrosylation is thought to inhibit contractile function; stimulatory effects are held to be the result of NO‐dependent soluble guanyl cyclase (sGC)/cyclic GMP (cGMP)/protein kinase G (PKG) signaling (Maréchal and Gaily 1999). A direct effect of NO_2_^−^ would therefore seemingly not explain the positive correlation we observed between changes in plasma NO_2_^−^ and changes in Pmax. Rather, this observation is consistent with our first hypothesis that interindividual differences in the availability of NO itself contribute to interindividual differences in the extent to which dietary NO_3_^−^ intake increases muscle power.

Although we were able to at least partially confirm our first hypothesis, our data do not support our second hypothesis, which was that individuals with a greater percentage of fast‐twitch fibers would demonstrate a greater dietary NO_3_^−^‐induced increase in muscle power. In particular, we found no correlation between the increase in Pmax and Vmax or fatigue index during the placebo trial. Although indirect, numerous previous studies have demonstrated that these (or comparable) measurements are significantly correlated with muscle fiber type (e.g., Coyle et al. 1979; Ivy et al. 1981; McCartney et al. 1983). Moreover, *V*_max_ and fatigue index were highly characteristic of a given subject, as indicated their high intraclass correlation coefficients. Given the strength of the association between muscle fiber type, speed, and fatigability found in previous studies (Coyle et al. 1979; Ivy et al. 1981; McCartney et al. 1983) along with the reproducibility of our measurements and the >2‐fold range in *V*_max_ and fatigue index during the placebo trial, it seems unlikely that the lack of correlation of the latter measures with the magnitude of the increase in Pmax with NO_3_^−^ ingestion is the result a type II statistical error. On the other hand, the premise that dietary NO_3_^−^ supplementation selectively targets fast‐twitch fibers is based largely on animal studies of muscle blood flow and oxygenation during aerobic exercise, for example, (Ferguson et al. 2015), and is only indirectly supported by human data. Specifically, Bailey et al. (2015) demonstrated dietary NO_3_^−^‐induced differences in muscle oxygenation, whole‐body VO_2_ kinetics, and performance during cycling when pedaling at 115 rpm but not at 35 rpm. Breese et al. (2013) reported similar benefits during the transition from moderate to high‐intensity exercise but not from low to moderate intensity exercise. These data, along with the fact that we have previously reported that NO_3_^−^ improves muscle function only at higher velocities (Coggan et al. 2015a,b), have been interpreted by Jones et al. (2016) as reflecting enhanced recruitment of fast‐twitch fibers at a higher velocities/intensities of exercise. It is unclear, however, whether altering pedaling rate in fact changes the pattern of motor unit recruitment (Ahlquist et al. 1992). Similarly, the relationship between exercise intensity and O_2_ flux is complex, with motor unit recruitment being only one influencing factor (Jones et al. 2011). Finally, in both fast‐ *and* slow‐twitch muscle NO seems to improve contractile function by increasing the rate of cross‐bridge cycling, not the amount of force per cross‐bridge (Maréchal and Gaily 1999). The resultant shift in the force‐velocity (and hence power‐velocity) curve, and not a selective impact only in fast‐twitch fibers, may therefore explain why we have previously observed statistically significant NO_3_^−^‐induced improvements in power only at higher speeds of contraction (Coggan et al. 2015a,b). The notion that dietary NO_3_^−^ affects only, or even primarily, human fast‐twitch fibers would therefore still seem equivocal.

An unexpected observation in this study was that, at least in terms of improvements in Pmax, women seem to benefit more than men from dietary NO_3_^−^ intake. Specifically, although not significant in the univariable analyses, female sex was the only predictor other than plasma NO_2_^−^ concentration selected by the stepwise forward regression procedure. Female subjects also tended to be more to likely be “high responders” to NO_3_^−^ supplementation, with five out of seven demonstrating greater‐than‐average increases in *P*_max_. This was true even though NO_3_^−^ intake increased plasma NO_2_^−^ concentration similarly in both women (i.e., +131 ± 162%) and men (i.e., +124 ± 93%). Previous studies of the effects of dietary NO_3_^−^ on exercise performance have included only male subjects (e.g., Christensen et al. 2013; Boorsma et al. 2014; Haider and Folland 2014; Hoon et al. 2014; Bailey et al. 2015; Whitfield et al. 2017), or have not commented on possible sex‐related differences (e.g., Breese et al. 2013; Hoon et al. 2015). It has been reported, however, that plasma NO_3_^−^ (Jilma et al. 1996; Ghasemi et al. 2008) and/or breath NO levels (Jilma et al. 1996; Olivieri et al. 2006) are lower in women. The reason for this difference is not known, but it may be due to suppression of NO production by progesterone (Scichilone et al. 2013) and/or a sex‐related difference in the distribution of a polymorphism in the neuronal NO synthase (NOS) gene (Grasemann et al. 2003). Regardless, lower NO bioavailability under baseline conditions could explain why the women seemed to be more responsive to dietary NO_3_^−^ intake. Indeed, we have previously observed an approximately twofold greater dietary NO_3_‐induced improvement in muscle power in patients with HF (Coggan et al. 2015a) compared to healthy control subjects (Coggan et al. 2015b) or athletes (Rimer et al. 2016), presumably because of diminished NOS‐mediated NO production (Katz et al. 1999) and enhanced NO destruction (Münzel et al. 2015) in patients with HF. Somewhat along the same lines, Kapil et al. (2010) found that changes in blood pressure in response to NO_3_^−^ ingestion were greatest in individuals with lower baseline plasma NO_2_^−^ concentrations (and higher baseline blood pressures), although in this case it was men who benefited the most. In any case, future studies should more directly address possible sex‐related differences in the effects of NO_3_^−^ ingestion on exercise performance.

There are a number of limitations to this study. The most obvious is that muscle biopsies were not performed to directly determine fiber type distribution, which potentially could have revealed a relationship between the percentage of fast‐twitch fibers and the relative increase in Pmax. Our study also included a relatively small number of individuals, only two of which were regular exercisers and none of whom were presently competing in endurance sports. Whether similar results would be obtained in a larger group of subjects and/or among athletes therefore cannot be determined from the present data. Although we used a randomized, placebo‐controlled, cross‐over design, there were no significant order effects, and *P*_max_, *V*_max_, and fatigue index proved to be highly reliable, it is possible that inclusion of a familiarization trial would have altered the results (especially in the several subjects in whom NO_3_^−^ ingestion seemed to impair muscle function.

Finally, although we have been able to identify two factors (i.e., plasma NO_2_^−^ concentration and possibly subject sex) contributing to interindividual differences in the effects of dietary NO_3_‐ on muscle contractile function, it must be emphasized that over half of this variability remains unexplained. Of course, some of this variability represents normal day‐to‐day variation in human performance (Coggan and Costill 1984), and is not due to NO_3_^−^ ingestion per se. Such random variability, however, could not explain the wide range of responses we observed, and as indicated previously measurement of *P*_max_ was highly reproducible. Additional studies measuring NO_3_^−^ reduction in the mouth as well as NO/sGC/cGMP/PKG signaling in muscle may provide further insight into the mechanism(s) responsible for this marked interindividual variability in the effects of dietary NO_3_^−^ on muscle power.

In summary, in this study we sought to identify factors influencing the magnitude of the improvement in muscle power due to dietary NO_3_^−^ intake. Our findings indicate that variable increases in NO bioavailability, as indicated by changes in plasma NO_2_^−^ concentration, along with subject sex account for ~40% of this variability. On the other hand, interindividual differences in muscle fiber type do not appear to be important. Much of the variation in response between individuals remains unexplained.

## Conflict of Interest

None to declare.
